# Oral β-lactam combinations are effective *in vitro* against *Mycobacterium avium*, regardless of clarithromycin susceptibility

**DOI:** 10.1128/spectrum.02012-25

**Published:** 2025-09-26

**Authors:** Maiko Yoshikawa, Tomoyasu Nishimura, Kana Misawa, Rina Shimamura, Kenta Suzuki, Shoko Kashimura, Yuki Igarashi, Yuki Enoki, Kazuaki Taguchi, Naoki Hasegawa, Ho Namkoong, Kazuaki Matsumoto

**Affiliations:** 1 Division of Pharmacodynamics, Keio University Faculty of Pharmacy, Minato-ku, Tokyo, Japan; 2 Department of Infectious Diseases, Keio University School of Medicine, Shinjuku-ku, Tokyo, Japan; 3 Keio University Health Center, Shinjuku-ku, Tokyo, Japan; 4 Department of Pharmacy, Okayama University, Okayama-shi, Okayama, Japan; 5 Graduate School of Biomedical and Health Sciences, Hiroshima University, Hiroshima-shi, Hiroshima, Japan; JMI Laboratories, North Liberty, Iowa, USA

**Keywords:** nontuberculous mycobacteria, *Mycobacterium avium *complex, macrolide resistance, β-lactam, tebipenem

## Abstract

The global incidence and prevalence of pulmonary disease caused by the *Mycobacterium avium* complex (MAC), mainly comprising *M. avium* and *Mycobacterium intracellulare*, is increasing. However, treating MAC pulmonary disease is challenging in cases of clarithromycin (CLR)-resistant MAC or where the patients experience adverse effects or drug interactions with the few available antibiotics. Therefore, developing novel and highly effective antibiotics against MAC is crucial. Although the efficacy of dual β-lactams against *Mycobacterium abscessus* has been receiving attention, the efficacy of dual β-lactams against MAC remains unclear. Here, we used MAC type strains and clinical isolates to determine whether dual β-lactams were effective against MAC and which combinations synergistically inhibited bacterial growth using a broth microdilution checkerboard assay with 6 oral and 22 intravenous antibiotics. The combination effect and antibacterial activity differed between *M. avium* and *M. intracellulare*. Five combinations of oral β-lactams and 78 combinations of intravenous β-lactams showed a synergistic effect against the *M. avium* type strain. Among the *M. avium* clinical isolates, faropenem combined with cefuroxime showed the highest synergistic effect, and amoxicillin combined with tebipenem showed the lowest minimum inhibitory concentration. There was no significant difference in the combination effects between the CLR-susceptible and CLR-resistant *M. avium* clinical isolates in these pairs. In conclusion, regardless of CLR susceptibility, the oral β-lactam combinations were effective against *M. avium*. Thus, when treating MAC pulmonary disease, it is crucial to determine whether *M. avium* or *M. intracellulare* is the cause.

**IMPORTANCE:**

*Mycobacterium avium* complex causes chronic respiratory infections, but treatment is often limited by drug resistance, intolerance, or interactions. As new therapeutic strategies are urgently needed, we focused on β-lactam antibiotics, which are widely used and well tolerated. Although dual β-lactams are effective against *Mycobacterium abscessus*, their utility against *Mycobacterium avium* complex has remained largely unexplored. Our *in vitro* study revealed that several β-lactam combinations are effective against *Mycobacterium avium*, regardless of drug resistance, indicating potential for clinical use. In contrast, *Mycobacterium intracellulare* showed lower susceptibility to β-lactams. Given this difference in drug susceptibility, we emphasize the clinical need to distinguish *Mycobacterium avium* and *Mycobacterium intracellulare* to optimize treatment of *Mycobacterium avium* complex pulmonary disease.

## INTRODUCTION

The global incidence and prevalence of pulmonary disease caused by nontuberculous mycobacteria (NTM), including the *Mycobacterium avium* complex (MAC), is increasing (1, 2). The MAC is a group of slow-growing mycobacteria, mainly comprising *M. avium* and *Mycobacterium intracellulare*, and is the most common NTM worldwide (2–4).

The standard treatment for MAC pulmonary disease is combination chemotherapy comprising macrolides (clarithromycin [CLR] or azithromycin), ethambutol, and rifampicin, with streptomycin or amikacin added depending on the disease severity, which is assessed using chest imaging and quantification of the bacterial burden of isolates (5, 6). Macrolides are the main antibiotics used for treating MAC pulmonary disease. However, macrolide monotherapy is the most frequent cause of macrolide-resistant MAC (7), defined as CLR-resistant bacteria with a CLR minimum inhibitory concentration (MIC) ≥32 µg/mL (8). Although the sputum culture conversion rate for pulmonary disease due to CLR-susceptible MAC by the standard treatment is 53.3%–77.4% (9), that for pulmonary disease due to CLR-resistant MAC has decreased to 14%–30% (10). There is no established and effective treatment regimen for pulmonary disease due to CLR-resistant MAC, and it has a poor prognosis. Ethambutol, rifampicin, and aminoglycosides are also typically administered for MAC pulmonary disease. Ethambutol (11) and aminoglycosides (12) cause optic and auditory neuropathy, respectively. Rifampicin induces cytochrome P450 enzymes, significantly reducing the plasma concentration of other drugs metabolized by these enzymes (13). Treatment for patients who are infected with CLR-resistant MAC or have adverse drug reactions or drug interactions is challenging because few antibiotics are available. Therefore, developing novel and highly effective antibiotics against MAC, especially CLR-resistant MAC, is crucial.

The development of new drugs is a lengthy and costly process. Drug repositioning is an approach in which a drug that is approved or in late-phase clinical trials is repurposed to treat diseases other than those for which it was initially intended, enabling a quicker provision of novel disease therapies. β-Lactams are commonly used in clinical practice, so their safety profile in humans has been established. Recently, the efficacy of dual β-lactam therapy against *Mycobacterium abscessus* has been reported *in vitro* and *in vivo* (14–19). The mechanism of action of this combination involves targeting enzymes, known as penicillin-binding proteins (PBPs). Similar to *M. abscessus*, the MAC genome encodes several PBPs (20). Thus, we hypothesized that dual β-lactams might also demonstrate a synergistic effect against MAC. There have been two reports on the efficacy of dual β-lactams against MAC. Negatu et al. evaluated the efficacy of nine oral β-lactam pairs against MAC, revealing that the combination of cefuroxime and tebipenem or sulopenem acted synergistically against *M. avium* (21). Al-Jabri et al. reported that combining meropenem with ceftaroline, cefdinir, or cefuroxime significantly reduced the MIC of meropenem (22). However, it remains unclear which β-lactam combinations are the most effective against MAC because these studies used only a few β-lactam combinations. Furthermore, PBPs differ between bacterial species (23). Since reports on the structure and interaction of PBPs with each β-lactam in MAC are scarce, it is difficult to predict the combined effects of β-lactams. Therefore, a comprehensive evaluation of the β-lactams used in clinical practice is necessary to identify favorable combinations.

Thus, this study aimed to determine whether dual β-lactams were effective against MAC. We also attempted to identify the dual β-lactams that exerted a synergistic effect against MAC type strains and validated their antibacterial activity against clinically isolated MAC, including CLR-resistant strains.

## RESULTS

### Oral β-lactam combinations showed synergistic effects against the *M. avium* type strain but not against the *M. intracellulare* type strain

Before performing the main experiments, we measured the MICs of three β-lactams (penicillin G, cefazolin, and tebipenem) alone or in combination with various β-lactamase inhibitors against *M. avium* ATCC700898 and *M. intracellulare* ATCC13950 (Table S1). For both strains, the MICs of penicillin G, cefazolin, and tebipenem were not reduced in combination with any of the tested β-lactamase inhibitors (8 µg/mL). However, sulbactam and tazobactam showed MICs of 64 µg/mL against *M. avium* ATCC700898, which was higher than the MICs of the three tested β-lactams. Thus, we did not add β-lactamase inhibitors to all combinations in the subsequent experiments.

**Fig 1:**
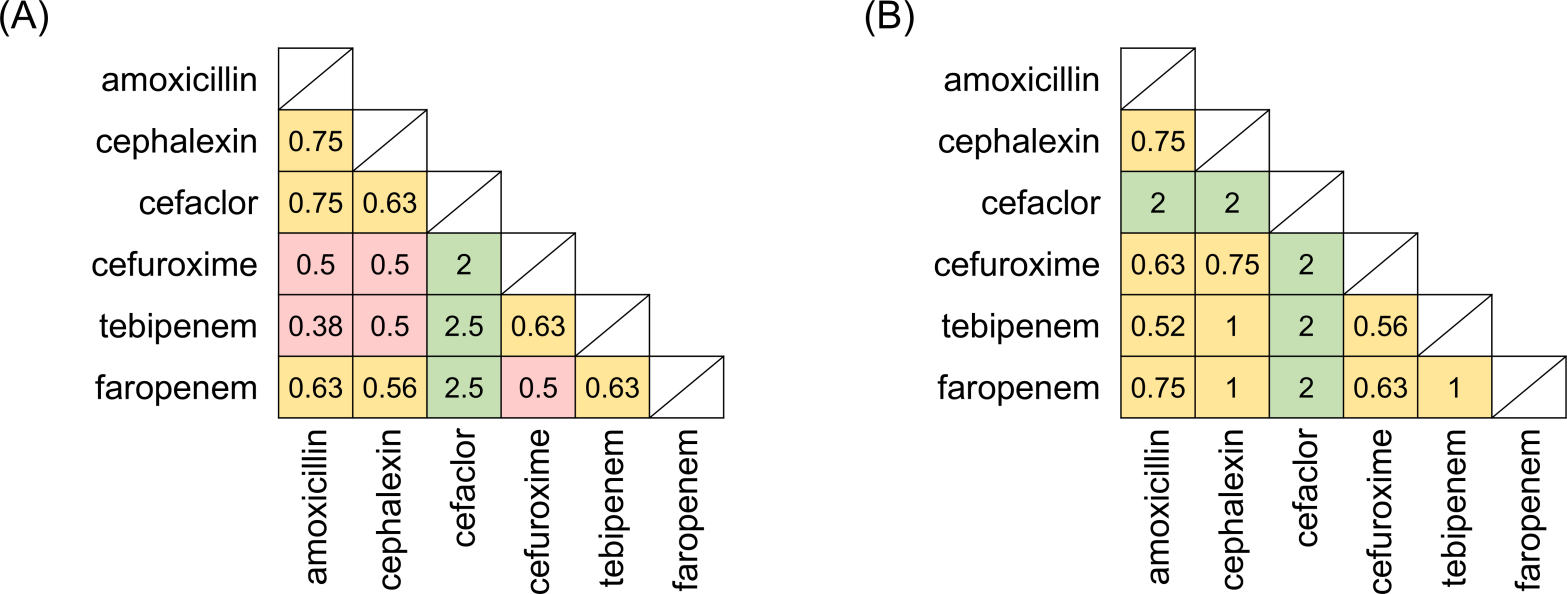
FIC indexes of oral β-lactam combinations against (**A**) *M. avium* type strain ATCC700898 and (**B**) *M. intracellulare* type strain ATCC13950. The MICs were determined by broth microdilution checkerboard assay and used to calculate the FIC indexes. Squares represent each β-lactam combination, with the two components indicated on the *x*- and *y*-axes, respectively. The number inside the square represents the respective FIC index value. Pink: FIC index ≤ 0.5, synergistic; yellow: 0.5 < FIC index ≤ 1.0, additive; green: 1.0 < FIC index ≤ 4.0, indifference. Untested combinations are shaded.

We first evaluated dual β-lactam efficacy using combinations of six oral β-lactams by checkerboard assay against MAC type strains (Fig. 1). Five combinations showed synergistic effects against *M. avium* ATCC700898 (Fig. 1A). Table 1 shows the MICs of these antibiotics alone and in combination. The combination of tebipenem and amoxicillin had the lowest fractional inhibitory concentration (FIC) index (0.38) and the lowest MICs (2 and 0.5 µg/mL, respectively). None of the pairs of oral β-lactams showed a synergistic effect against *M. intracellulare* ATCC13950 (Fig. 1B). The MICs of oral β-lactams against *M. intracellulare* ATCC13950 were higher than those against *M. avium* ATCC700898 (Table S2).

**TABLE 1:** MICs of the combination of two oral β-lactams that showed synergy against *M. avium* type strain ATCC70089^*a*^

β-Lactam A	MIC of β-lactam A (μg/mL)		β-Lactam B	MIC of β-lactam B (μg/mL)		FIC index
	Alone	With β-lactam B		Alone	With β-lactam A	
Cefuroxime	8	2	Amoxicillin	4	1	0.5
Cefuroxime	8	2	Cephalexin	8	2	0.5
Cefuroxime	8	2	Faropenem	16	4	0.5
Tebipenem	8	2	Amoxicillin	4	0.5	0.38
Tebipenem	8	2	Cephalexin	8	2	0.5

^*a*^ A checkerboard assay was performed within the concentration of β-lactams except for amoxicillin, which was 0.25 to 256 μg/mL, and that of amoxicillin, which was 0.125 to 128 μg/mL due to its solubility.

### Intravenous β-lactam combinations showed synergistic effects against MAC type strains

**Fig 2:**
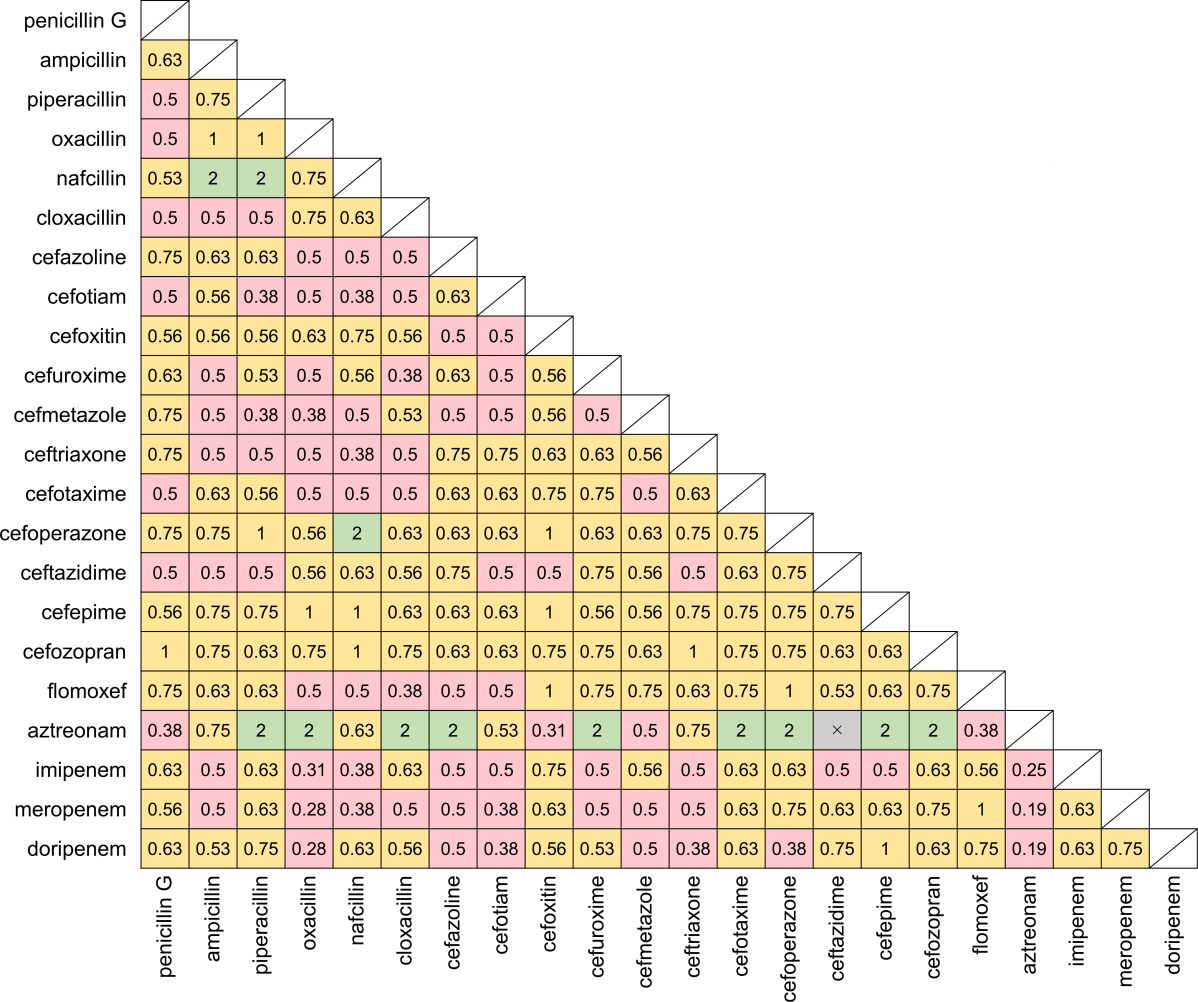
FIC indexes of the combinations of intravenous β-lactams against *M. avium* ATCC700898. Each square represents a combination of two β-lactams, with the two components indicated on the *x*- and *y*-axes, respectively. The number inside the square represents the respective FIC index value. Pink: FIC index ≤ 0.5, synergistic; yellow: 0.5 < FIC index ≤ 1.0, additive; green: 1.0 < FIC index ≤ 4.0, indifference. Crosses indicate that the MIC was not measurable within the tested concentrations. The concentrations of intravenous β-lactams were 0.25 µg/mL–256 µg/mL, except for cefoxitin, which was 0.125 µg/mL–128 µg/mL due to its solubility. Untested combinations are shaded.

**Fig 3:**
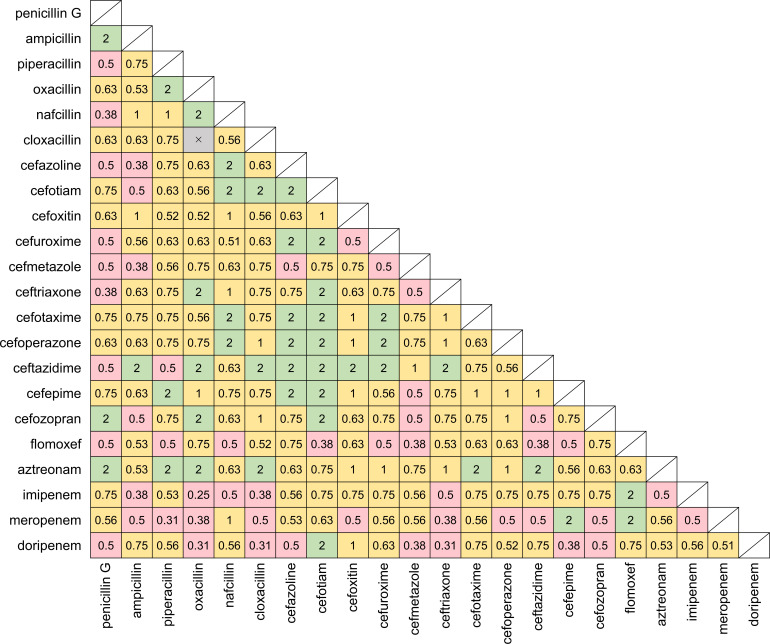
FIC indexes of the combinations of intravenous β-lactams against *M. intracellulare* ATCC13950. Each square represents a combination of two β-lactams, with the two components indicated on the *x*- and *y*-axes, respectively. The number inside the square represents the respective FIC index value. Pink: FIC index ≤ 0.5, synergistic; yellow: 0.5 < FIC index ≤ 1.0, additive; green: 1.0 < FIC index ≤ 4.0, indifference. Crosses indicate that the MIC was not measurable within the tested concentrations. The concentrations of intravenous β-lactams were 0.25 µg/mL–256 µg/mL, except for cefoxitin, which was 0.125 µg/mL–128 µg/mL due to its solubility. Untested combinations are shaded.

Next, we evaluated the efficacy of combinations of 22 intravenous β-lactams against MAC type strains using a checkerboard assay. Synergistic effects were observed against *M. avium* ATCC700898 for 78 combinations (Fig. 2). The combination of aztreonam with doripenem or meropenem showed the lowest FIC index, but the MICs of aztreonam remained high (32 µg/mL), even after combination (Table S3). The lowest MICs were 1 µg/mL–2 µg/mL, comparable to the MICs of oral β-lactams. Synergistic effects were observed against *M. intracellulare* ATCC13950 for 51 intravenous combinations (Fig. 3). Of these combinations, 24 included intravenous carbapenems (imipenem, meropenem, or doripenem) (Table 2). The combination of imipenem and oxacillin showed the lowest FIC index, but the MIC of oxacillin was notably high (64 µg/mL). The synergistic combinations of ceftriaxone with doripenem or meropenem showed the lowest MICs (2 µg/mL–8 µg/mL). The MICs of intravenous β-lactams against *M. intracellulare* ATCC13950 were higher than those against *M. avium* ATCC700898 (Table 2; Tables S3 and S4).

**TABLE 2:** MICs of the intravenous combination of a carbapenem and other β-lactam that showed synergy against *M. intracellulare* type strain ATCC13950^*a*^

β-Lactam A	MIC of β-lactam A (μg/mL)		β-Lactam B	MIC of β-lactam B (μg/mL)		FIC index
	Alone	With β-lactam B		Alone	With β-lactam A	
Meropenem	32	8	Ampicillin	64	16	0.5
Meropenem	32	2	Piperacillin	256	64	0.31
Meropenem	32	8	Oxacillin	>256	64	0.38
Meropenem	32	8	Cloxacillin	>256	128	0.5
Meropenem	32	8	Cefoxitin	128	32	0.5
Meropenem	32	8	Ceftriaxone	32	4	0.38
Meropenem	32	8	Cefoperazone	128	32	0.5
Meropenem	32	8	Ceftazidime	128	32	0.5
Meropenem	32	8	Cefozopran	32	8	0.5
Meropenem	32	8	Imipenem	64	16	0.5
Doripenem	32	8	Penicillin G	128	32	0.5
Doripenem	32	2	Oxacillin	>256	128	0.31
Doripenem	32	2	Cloxacillin	>256	128	0.31
Doripenem	32	8	Cefazolin	64	16	0.5
Doripenem	32	4	Cefmetazole	128	32	0.38
Doripenem	32	2	Ceftriaxone	32	8	0.31
Doripenem	32	4	Cefepime	64	16	0.38
Doripenem	32	8	Cefozopran	32	8	0.5
Imipenem	64	8	Ampicillin	64	16	0.38
Imipenem	64	8	Oxacillin	>256	64	0.25
Imipenem	64	16	Nafcillin	64	16	0.5
Imipenem	64	16	Cloxacillin	>256	64	0.38
Imipenem	64	16	Ceftriaxone	32	8	0.5
Imipenem	64	16	Aztreonam	256	64	0.5

^*a*^ A checkerboard assay was performed within the concentration of β-lactams except for cefoxitin, which was 0.25 to 256 μg/mL, and that of cefoxitin was 0.125 to 128 μg/mL due to its solubility.

### MICs of oral β-lactam therapy against *M. avium* clinical isolates

We observed a synergistic effect against the *M. avium* type strain for 5 combinations of oral β-lactams and 78 combinations of intravenous β-lactams. To confirm the efficacy of oral β-lactam combinations for treating outpatients with *M. avium* pulmonary disease, we measured the MICs of oral β-lactams against the 56 clinical isolates (CLR-susceptible, *n* = 29; CLR-resistant, *n* = 27) and calculated the MIC_50_ and the MIC_90_ values and the FIC indexes.

Table 3 and Table S5 list the MICs of five oral β-lactams (amoxicillin, cephalexin, cefuroxime, tebipenem, and faropenem) alone against CLR-susceptible and CLR-resistant *M. avium* strains. The MIC_50_ and MIC_90_ values against the CLR-resistant *M. avium* strains were almost twofold higher than those against the CLR-susceptible *M. avium* strains. Furthermore, the range of MIC values against the CLR-resistant *M. avium* strains was wider than that against the CLR-susceptible *M. avium* strains. Table 4 and Table S6 show the MICs of five oral β-lactam combinations (cefuroxime with amoxicillin, cefuroxime with cephalexin, cefuroxime with faropenem, tebipenem with amoxicillin, and tebipenem with cephalexin) against the *M. avium* clinical strains. Against CLR-susceptible isolates, the MIC_50_ and MIC_90_ values of tebipenem were reduced from 4/8 to 1/4 µg/mL in combination with amoxicillin or cephalexin, while the MIC_50_ and MIC_90_ values of amoxicillin or cephalexin were also reduced from 8/16 to 2/8 and 2/4 µg/mL, respectively. Such decreases in the MICs were also observed against CLR-resistant isolates, with the MIC_50_ and MIC_90_ values of tebipenem reduced from 8/16 to 2/8 µg/mL in combination with 4/8 µg/mL of amoxicillin. Although the MIC_50_ and MIC_90_ values of cephalexin were 8/16 µg/mL, those of tebipenem were reduced to 1/8 µg/mL. The MICs of cefuroxime were also reduced in the combinations but were inferior to those of tebipenem.

**TABLE 3:** MICs of oral β-lactams alone against *M. avium* clinical strains^*a*^

β-Lactam	MIC (μg/mL)					
	CLR-susceptible (*n* = 29)			CLR-resistant (*n* = 27)		
	Range	MIC_50_	MIC_90_	Range	MIC_50_	MIC_90_
Amoxicillin	2–32	8	16	0.5–128	8	32
Cephalexin	2–64	8	16	1–256	16	64
Cefuroxime	2–64	8	32	1–256	16	64
Tebipenem	0.5–16	4	8	0.25–32	8	16
Faropenem	1–128	16	64	0.5–128	32	64

^*a*^ CLR-susceptible: *n* = 29, CLR-resistant: *n* = 27. MIC_50/90_, the concentration inhibiting 50/90% of the strains.

**TABLE 4:** MICs of oral β-lactam combinations against *M. avium* clinical strains^*a*^

β-Lactam A	MIC of β-lactam A (μg/mL)						β-Lactam B	MIC of β-lactam B (μg/mL)					
	CLR-susceptible (*n* = 29)			CLR-resistant (*n* = 27)				CLR-susceptible (*n* = 29)			CLR-resistant (*n* = 27)		
	Range	MIC_50_	MIC_90_	Range	MIC_50_	MIC_90_		Range	MIC_50_	MIC_90_	Range	MIC_50_	MIC_90_
Cefuroxime	0.5–16	2	8	0.25–32	4	16	Amoxicillin	0.25–8	2	8	0.25–16	4	8
Cefuroxime	0.25–8	2	4	0.5–32	4	16	Cephalexin	0.5–16	2	8	0.25–128	4	32
Cefuroxime	0.5–16	4	8	0.25–32	4	16	Faropenem	0.5–32	4	8	0.063–32	4	16
Tebipenem	0.125–8	1	4	0.016–8	2	8	Amoxicillin	0.5–16	2	8	0.25–32	4	8
Tebipenem	0.25–8	1	4	0.063–16	1	8	Cephalexin	0.25–16	2	4	0.125–32	8	16

^*a*^ CLR-susceptible: *n* = 29, CLR-resistant: *n* = 27. MIC_50/90_, the concentration inhibiting 50/90% of the strains.

### Combinations of two oral β-lactams were effective against *M. avium* clinical isolates regardless of CLR susceptibility

**Fig 4:**
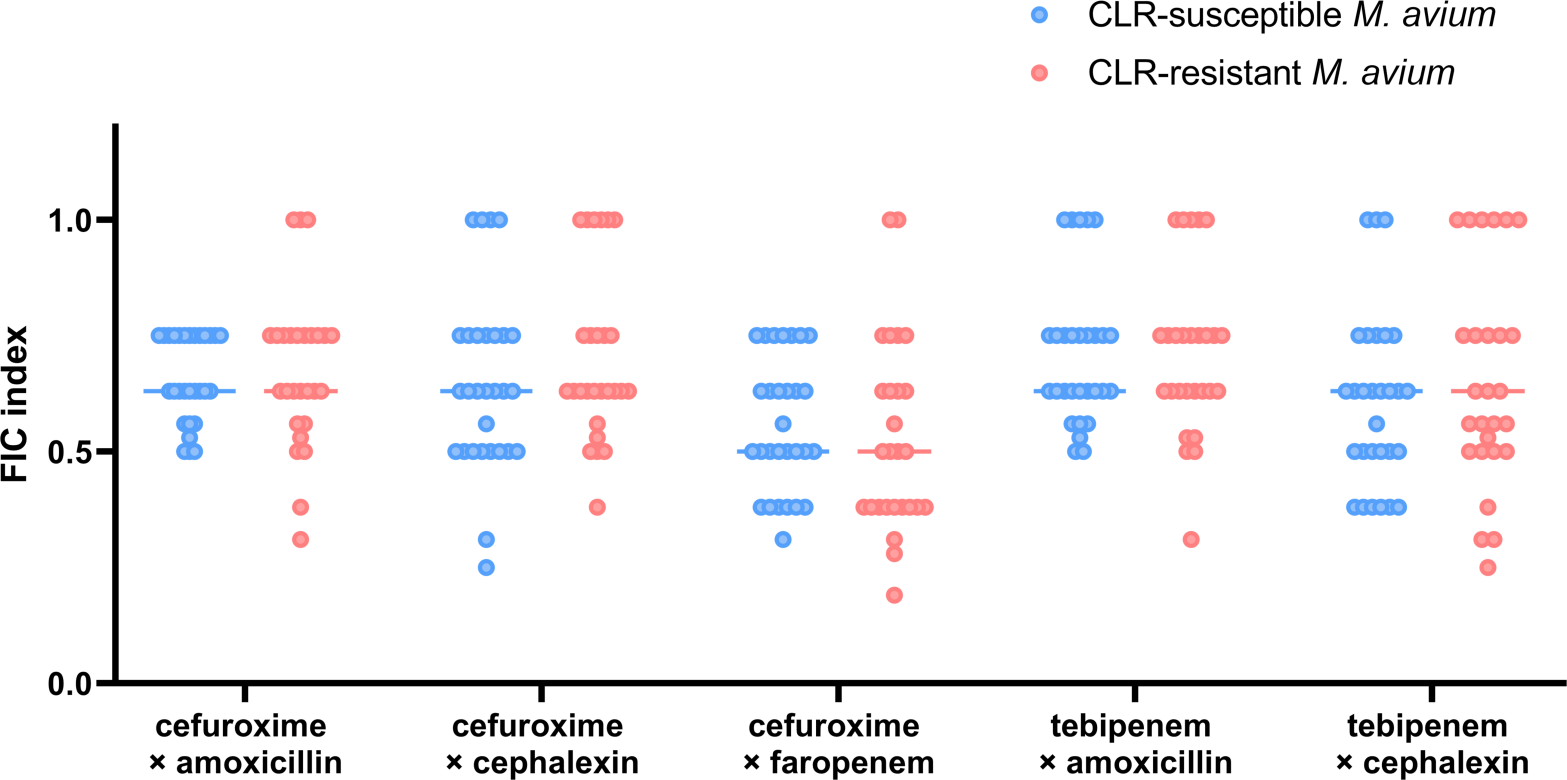
FIC indexes of oral β-lactam combinations against CLR-sensitive (*n* = 29) and CLR-resistant (*n* = 27) *M. avium* clinical isolates. Each point indicates the FIC index value for each strain, and each bar shows the median of the FIC indexes. Blue points and bars: CLR-sensitive strains. Pink points and bars: CLR-resistant strains. FIC index ≤ 0.5, synergistic and 0.5 < FIC index ≤1.0, additive.

The MICs of five oral β-lactam combinations against 29 CLR-susceptible and 27 CLR-resistant *M. avium* clinical isolates (Table S7) were used for calculating the FIC indexes (Fig. 4). These ranged from 0.19 to 1.0 among the clinical isolates of *M. avium* strains, indicating that the oral β-lactam combinations showed synergistic or additive effects against all the clinical isolates. The number of strains against which each combination showed a synergistic effect differed. The combination of cefuroxime and faropenem showed a synergistic effect in 15/29 (52%) CLR-susceptible strains and 16/27 (59%) CLR-resistant strains, while the combination of tebipenem and amoxicillin showed a synergistic effect in 2/29 (7%) CLR-susceptible strains and 3/27 (11%) CLR-resistant strains. In all combinations, there was no significant difference in the FIC indexes between the CLR-susceptible and CLR-resistant strains (cefuroxime with amoxicillin, *P* = 0.75; cefuroxime with cephalexin, *P* = 0.29; cefuroxime with faropenem, *P* = 0.32; tebipenem with amoxicillin, *P* = 0.90; and tebipenem with cephalexin, *P* = 0.48).

### Intravenous β-lactam combinations showed a synergistic effect but a high MIC against *M. intracellulare* clinical isolates

None of the combinations of oral β-lactams showed synergistic effects against the *M. intracellulare* type strain. In contrast, 51 combinations of intravenous β-lactams showed synergistic effects against the *M. intracellulare* type strain. Using 25 CLR-susceptible and 4 CLR-resistant *M. intracellulare* clinical isolates, we calculated the MIC_50_ and MIC_90_ values and the FIC indexes of the identified combinations (ceftriaxone with doripenem or meropenem). Against the CLR-susceptible *M. intracellulare*, the MIC_50_ and the MIC_90_ values of ceftriaxone, doripenem, and meropenem alone were all 64/128 µg/mL (Table S8). When ceftriaxone was used in combination with doripenem or meropenem, the MIC_50_ and MIC_90_ values of ceftriaxone were 16/64 µg/mL, and those of doripenem and meropenem were 8/32 µg/mL (Table 5; Table S9). Table S10 shows the MICs used to calculate the FIC indexes. Both combinations exhibited FIC indexes of 0.31–0.75, indicating synergistic or additive (Fig. S1) effects. The sample size was too small to calculate the statistical values for CLR-resistant *M. intracellulare*, but the MICs and the FIC indexes were close to the distribution of CLR-susceptible strains (Table S10; Fig. S1).

**TABLE 5:** MICs of intravenous β-lactam combinations against CLR-susceptible *M. intracellulare* clinical strains^*a*^

β-Lactam A	MIC of β-lactam A (μg/mL)			β-Lactam B	MIC of β-lactam B (μg/mL)		
	Range	MIC_50_	MIC_90_		Range	MIC_50_	MIC_90_
Ceftriaxone	2–128	16	64	Doripenem	0.125–64	8	32
Ceftriaxone	2–128	16	64	Meropenem	0.125–64	8	32

^*a*^ *n* = 25. MIC_50/90_, the concentration inhibiting 50/90% of the strains.

## DISCUSSION

This study identified dual combinations of clinically available β-lactams that were effective against *M. avium*, including CLR-resistant strains. We also observed notable differences between *M. avium* and *M. intracellulare* regarding the impact of dual β-lactam combinations and the MIC distribution. These findings indicate the importance of determining whether *M. avium* or *M. intracellulare* is the cause of MAC pulmonary disease so that appropriate treatment can be given.

It was reported that MAC clinical isolates did not produce β-lactamase (24–26). To confirm this, we investigated whether the MICs of β-lactams were reduced by combinations of β-lactamase inhibitors against MAC type strains, revealing that they remained unchanged (Table S1). Some studies have reported that avibactam did not enhance benzylpenicillin, ceftriaxone, and ertapenem activity against *M. avium* (27–29). We confirmed that β-lactamase inhibitors did not improve β-lactam activity against MAC. We also observed that high concentrations of sulbactam and tazobactam, which have a β-lactam ring structure, showed antibacterial activity against the *M. avium* type strain. Because sulbactam inhibits *Acinetobacter baumannii* growth by binding to PBP1 and PBP3 (30), it is plausible that sulbactam and tazobactam directly interact with PBPs of *M. avium*. Notably, no such antibiotic activity was observed against *M. intracellulare*.

The checkerboard assay of oral β-lactams revealed five synergistic pairs, all of which included cefuroxime or tebipenem, against *M. avium* ATCC700898. However, none of the pairs showed a synergistic effect against *M. intracellulare* ATCC13950 (Fig. 1). Compared with the findings of Negatu et al. (21), the degree of the combination effects observed in our study was small, although synergistic effects were shown with combinations of cefuroxime, tebipenem, or amoxicillin against *M. avium*. This discrepancy in the results may be due to the *M. avium* strains or the culture medium composition. Notably, the MICs of mycobacteria were reported to differ depending on whether Middlebrook 7H9 broth or cation-adjusted Mueller-Hinton broth (CAMHB) was used for broth microdilution (31–33). For oral drug pairs, our findings agreed with Negatu et al., who reported that the combined effects for *M. intracellulare* were less than those for *M. avium* (21). When intravenous β-lactams were combined, synergistic effects against both *M. avium* ATCC700898 and *M. intracellulare* ATCC13950 were observed (Fig. 2 and 3). Al-Jabri et al. reported that three combinations of meropenem with cephalosporins were effective against MAC (22), and our study also showed some synergistic pairs of carbapenems and cephalosporins, including carbapenems with early-generation cephalosporins against *M. avium* and carbapenems with later-generation cephalosporins against *M. intracellulare*. These results suggest that the cell wall structure and PBP profile differ between *M. avium* and *M. intracellulare*.

The mechanism by which combinations showed a synergistic effect is based on the insight that β-lactams target different PBPs involved in peptidoglycan synthesis. An example of dual β-lactam therapy based on this mechanism is the clinical use of the combination of ampicillin and ceftriaxone against *Enterococcus faecalis* (34–37). PBPs are peptidoglycan synthetases. They are classified as D,D-transpeptidases (Ddts), L,D-transpeptidases (Ldts), and D,D-carboxypeptidases (23). Ddts and Ldts cross-link peptidoglycans with a 4′−3′ or 3′−3′ bond of stem peptides, respectively (38). D,D-carboxypeptidases are important for producing Ldt substrates (38). Ddts are inactivated by almost all β-lactams, whereas Ldt inhibition is limited to carbapenems and penems and to a lesser degree by cephalosporins (39–43). Compared with the general predominance of Ddt in bacteria, Ldts are responsible for most of the peptidoglycan cross-linking in *Mycobacterium tuberculosis* and *M. abscessus* (44, 45). Thus, carbapenems capable of inactivating Ldts are important to overcome the resistance of *M. tuberculosis* and *M. abscessus* to β-lactams. Investigation of the predominance and the percentage of 3′−3′ bonds in MAC cell wall peptidoglycan is necessary, as is that of other mycobacteria. The MAC genome encodes several subclasses of Ddts, Ldts, and D,D-carboxypeptidases, as does the genome of *M. tuberculosis* and *M. abscessus* (20, 46). In *M. avium*, Ldt_Mav2_ was inhibited by faropenem, tebipenem, imipenem, doripenem, biapenem, and cephalothin but not by amoxicillin, ampicillin, cefoxitin, or meropenem (40). In our study, tebipenem with amoxicillin and tebipenem with faropenem showed synergistic and additive effects, respectively (Fig. 1A). Thus, the mechanism by which the combination is synergistic against *M. avium* may involve a β-lactam that inhibits Ldt_Mav2_, making up for the lack of inhibition of Ldt_Mav2_ by another β-lactam. On the other hand, cefuroxime showed a synergistic effect with faropenem but an additive effect with tebipenem, suggesting that the mechanism cannot only be explained by Ldt_Mav2_ inhibition. It is thought that multiple PBPs play important roles in the synergistic effects of β-lactam antibiotics on MAC and in MAC survival and growth mechanisms. However, it is difficult to determine the mechanism of dual β-lactams against *M. intracellulare* because there are no studies on its PBPs. We observed the synergistic effect of the combination of carbapenems with cephalosporins against *M. intracellulare*, suggesting the combined effect against *M. intracellulare* was achieved by inhibiting multiple PBPs, as with *M. avium*. Furthermore, although it is generally accepted that β-lactams of the same family, such as penicillins, cephalosporins, or carbapenems, inhibit the same PBPs (23), a study has reported that each β-lactam within the same family binds to different PBPs (40). In our study, the same family combinations, such as cephalexin and cefuroxime, also showed synergistic effects (Fig. 2 to 4). It has not been demonstrated whether these β-lactams inhibit the same PBPs or show different inhibitory patterns. In *M. abscessus*, the combination of imipenem and ceftaroline showed a synergistic effect despite overlapping targets, including Ldt_Mab1_, Ldt_Mab2_, and Ldt_Mab4_ (18). Because imipenem had a higher affinity to these targets than ceftaroline, the authors hypothesized that imipenem was the first to bind to the targets, changing the conformation to facilitate ceftaroline binding to the same target. Although they were able to support this hypothesis *in silico*, it required flexibility of the active site. In short, to reveal the mechanism of β-lactam combination effects against MAC, further basic studies are necessary to characterize the PBP molecules of MAC and analyze the binding affinities and conformations between the PBPs and each β-lactam.

The oral combination of tebipenem and amoxicillin against *M. avium* ATCC700898 had the lowest MICs, reduced to 2 and 0.5 µg/mL, respectively (Table 1). It was suggested that these concentrations could be reached or were close to being achieved in the epithelial lining fluid (ELF) in clinically healthy adults (47, 48). Tebipenem pivoxil hydrobromide administration (five doses of 600 mg every 8 h) resulted in an ELF concentration of 0.824 µg/mL (48). Furthermore, the administration of a single dose of amoxicillin (500 mg) resulted in a concentration of 0.89 µg/mL in ELF (47). Considering the long treatment period (years) for MAC pulmonary disease (5), oral drugs are favorable for outpatients.

The MICs of β-lactams against CLR-resistant *M. avium* were higher than those of CLR-susceptible strains (Tables 3 and 4). This may be related to the host environment. In our study, CLR-resistant strains had been exposed to several antibiotics, whereas CLR-susceptible strains were only included if they were from patients whose medical records showed no indication of antimicrobial treatment. The standard therapeutic drugs for MAC pulmonary disease (CLR, ethambutol, rifampicin, and amikacin) induce the upregulation of various transporter genes, including putative low-specificity transporters (49), suggesting that antibiotic efflux may cause the high MICs in CLR-resistant strains. However, CLR-resistant *M. avium* also showed the lowest MICs to β-lactams (Tables 3 and 4). To our knowledge, there are no reports of strains resistant to one class of antibiotics having a broader MIC range for another class than susceptible strains. It is unclear whether these highly sensitive strains were naturally susceptible or acquired the characteristics following antibiotic exposure. Thus, analyzing strains with different susceptibilities is key to understanding β-lactam resistance mechanisms. The FIC indexes between CLR-susceptible *M. avium* and CLR-resistant *M. avium* were not significantly different (Fig. 4), confirming the prediction of Negatu et al. (21) that β-lactam combinations were effective regardless of CLR sensitivity, thereby providing promising treatment choices for CLR-resistant *M. avium* pulmonary disease with limited treatment options.

Although the MIC distribution of β-lactams tended to be higher in *M. intracellulare* than in *M. avium*, the tested β-lactams differed (Table 4; Table S7). However, this difference may be caused by biological variations between the species. It is unlikely that there was any bias due to prior antibiotic exposure because CLR-susceptible MAC strains were exclusively collected from treatment-naïve cases. A possible explanation is that the susceptibility of β-lactams against MAC differs considerably among species, with *M. intracellulare* leaning toward resistance. This tendency is supported by a Korean report showing that the MIC distribution of the antimicrobial agents against NTM, including imipenem and cefoxitin (classed as β-lactams), was notably higher in *M. intracellulare* than in *M. avium* (50).

Because the breakpoints for β-lactams have not been defined against MAC, it was impossible to determine the susceptibility of β-lactams alone and in combination (8). Furthermore, the correlation between the *in vitro* susceptibility of β-lactams for MAC and the clinical response has not been studied. Thus, further *in vivo* studies and clinical trials are necessary to analyze the relationship between the MICs of β-lactams and the clinical outcome. Since most of the drugs we examined have already been approved, clinical trials are expected to begin soon. Although tebipenem is only approved for children in Japan, phase III trials of oral tebipenem among adults are underway to treat complicated urinary tract infections and acute pyelonephritis. In lung infections, the concentration of antibiotics in the ELF or alveolar macrophages may reflect the antibacterial activity in pneumonia (51). As mentioned above, a single dose (500 mg) of amoxicillin (47) and the administration of tebipenem pivoxil hydrobromide (five doses of 600 mg every 8 h) (48) resulted in a concentration of 0.89 and 0.824 µg/mL, respectively, in ELF. These drug doses also resulted in serum concentrations of 6.9 µg/mL (47) and plasma concentrations of 8.09 µg/mL (48), respectively. A single dose of cefuroxime (500 mg) led to an ELF concentration of 0.7 µg/mL and a serum concentration of 3.9 µg/mL (52). The penetration of oral β-lactams into ELF is typically reported as a ratio of 0.12–0.38 to the total plasma concentration (53). Despite the lack of data on lung pharmacokinetics, single-dose cephalexin (500 mg) (54) and faropenem administration (300 mg three times a day for 5 days) (55) were reported to result in a plasma concentration of 21.29 and 5.7 µg/mL, respectively, in healthy adults. Considering these plasma concentrations, it is assumed that cephalexin and faropenem will reach 2.6–8.1 and 0.68–2.2 µg/mL, respectively, in ELF if lung penetration is as high as possible. The MICs of β-lactam against *M. avium* clinical strains in our study were higher than these estimated ELF concentrations. Although their pharmacokinetics in patients with MAC pulmonary disease and the correlation between their MICs and clinical response are unclear, higher concentrations should be maintained in the lungs to achieve a clinical cure. One idea for improving local concentrations is modifying the drug delivery system. Similar to β-lactams, amikacin is hydrophilic. Thus, compared with intravenous administration, intracellular uptake was increased 274-fold within the alveolar macrophages when it was encapsulated in liposomes and inhaled (56).

This study had some limitations. First, the clinical isolates were collected from a single hospital. Therefore, our findings must be confirmed in a multicenter study on a large number of MAC strains with various clinical backgrounds and collected from different geographical settings. Second, we did not fully evaluate the antibacterial activity and the combination effects against CLR-resistant *M. intracellulare* because, compared with *M. intracellulare*, *M. avium* was predominant at the collection site. Further evaluation of more CLR-resistant strains of *M. intracellulare* is required. Third, the antibacterial activity may have been underestimated due to the instability of the tested drugs in the broth used in the microdilution checkerboard assay. Carbapenems easily degenerate in solution, so their concentrations may not have been maintained throughout the assay. Different evaluation systems must be applied to accurately evaluate the effects.

In conclusion, the combination effect of β-lactam and its antibacterial activity differed between *M. avium* and *M. intracellulare*. Faropenem combined with cefuroxime showed the highest synergistic effect, and amoxicillin combined with tebipenem showed the lowest MIC against *M. avium*. These combinations did not show any significant differences in effects between CLR-susceptible and CLR-resistant *M. avium*. The oral β-lactam combinations were effective against *M. avium*, while dual β-lactam combinations were ineffective against *M. intracellulare*.

## MATERIALS AND METHODS

### Bacterial strains

Type strains *M. avium* Chester ATCC700898 and *M. intracellulare* ATCC13950 were obtained from Brigham and Women’s Hospital (Boston, USA). Clinical MAC isolates (*n* = 85), comprising 56 *M*. *avium* and 29 *M*. *intracellulare* strains, were cultured from respiratory specimens collected from patients at Keio University Hospital (Tokyo, Japan) from 2014 to 2021. All strains were cultured on Middlebrook 7H11 agar plates (Becton, Dickinson, and Company, MD, USA) at 37°C under 5% CO_2_ for approximately 3 weeks. Bacteria isolated from resultant colonies were suspended in Middlebrook 7H9 broth (Becton, Dickinson, and Company) containing 10% (vol/vol) Middlebrook OADC Enrichment (Becton, Dickinson, and Company), 0.2% glycerol, and 0.05% Tween 80. The suspension was aliquoted into cryotubes and stored at −80°C.

### Antibiotics

We used 6 oral and 22 intravenous β-lactam antibiotics, 5 β-lactamase inhibitors, and CLR for drug susceptibility tests against MAC (Table S11). Moxifloxacin and linezolid were used for quality control. Tebipenem and nacubactam were obtained from Meiji Seika Pharma Co., Ltd. (Tokyo, Japan). Stock solutions of antibiotics other than CLR were dissolved in sterile water and stored at −80°C until immediately before use. CLR was prepared in methanol and stored in the same manner.

### Antimicrobial susceptibility test

MICs were determined using the broth microdilution test recommended by the Clinical and Laboratory Standards Institute guideline M24-A2. The medium comprised CAMHB (Becton, Dickinson, and Company) supplemented with 5% Middlebrook OADC Enrichment. Each antibiotic was diluted twofold in the medium, and 50 µL was injected into each well. Aliquots of bacteria were filtered through a 5 µm diameter filter and prepared to a turbidity of 0.5 McFarland standard in sterile phosphate-buffered saline. Then the bacterial suspension was diluted 1:100 with the medium, and 50 µL was inoculated into each well (final concentration: approximately 5 × 10^5^ CFU/mL) and incubated for 7 days at 37°C. After confirming bacterial growth in the drug-free control well, the MICs were determined. If bacterial growth was insufficient, MICs were re-evaluated at 10–14 days. The MIC was defined as the lowest drug concentration without visible bacterial growth. *M. avium* ATCC700898 was used for quality control, with MICs of 0.25–2 µg/mL for moxifloxacin and 4–16 µg/mL for linezolid. MIC_50_ and MIC_90_ values represented the concentration inhibiting 50% and 90% of the isolates, respectively.

### CLR sensitivity of clinical isolates

The CLR sensitivity phenotype of all clinical strains was determined by measuring the MICs as described above. The strains were classified as CLR-susceptible (MIC ≤8 µg/mL) or CLR-resistant (MIC ≥32 µg/mL).

### Determination of FIC indexes using the checkerboard assay

We used a checkerboard assay for evaluating antimicrobial susceptibility when two drugs were used in combination, testing both agents in twofold serial dilutions. MICs were determined after 7 or 10–14 days of incubation at 37°C. The findings were only considered valid if there was bacterial growth in the drug-free control well. To determine the combination effect of two β-lactams, the FIC index was calculated using the following formula: FIC index = (MIC of drug A in combination / MIC of drug A alone) + (MIC of drug B in combination / MIC of drug B alone). The FIC index value was calculated based on the combination of antibiotics that produced the greatest change from the individual antibiotic’s MIC. If the MIC was >256 µg/mL, the FIC index was calculated assuming that the MIC was 512 µg/mL. An FIC index ≤0.5 was judged as synergistic, 0.5 < FIC index ≤ 1.0 as additive, and 1.0 < FIC index ≤ 4.0 as indifference (57).

### Statistical analysis

All statistical analyses were performed using GraphPad Prism 8 (GraphPad Software, San Diego, CA, USA). FIC indexes were compared between CLR-susceptible and CLR-resistant strains using the Mann-Whitney U-test. *P*-values <0.05 were considered statistically significant.

## SUPPLEMENTAL MATERIAL

Table S1 to S11 and Figure S1 spectrum.02012-25-s0001.docx

ASM does not own the copyrights to Supplemental Material that may be linked to, or accessed through, an article. The authors have granted ASM a non-exclusive, world-wide license to publish the Supplemental Material files. Please contact the corresponding author directly for reuse.
