# Supplementary material for: Oral β-lactam combinations are effective in vitro against Mycobacterium avium, regardless of clarithromycin susceptibility
Source: Microbiol Spectr. 2025 Sep 26;13(11):e02012-25. doi: 10.1128/spectrum.02012-25 (PMC12584685; doi:10.1128/spectrum.02012-25)
Supplement: Table S1 to S11 and Figure S1 [file spectrum.02012-25-s0001.docx]

Table S1 MICs of β-lactams in combination with β-lactamase inhibitor or not and MICs of β-lactamase inhibitors

|  |  | MIC (µg/mL) |  |
| --- | --- | --- | --- |
| drug |  | *M. avium*  ATCC700898 | *M. intracellulare* ATCC13950 |
| β-lactams |  |  |  |
| penicilliin G | alone | 4 | 128 |
|  | + clavulanic acid | 4 | 128 |
|  | + sulbactam | 4 | 128 |
|  | + tazobactam | 4 | 128 |
|  | + avibactam | 8 | 128 |
|  | + nacubactam | 8 | 128 |
| cefazolin | alone | 4 | 128 |
|  | + clavulanic acid | 4 | 128 |
|  | + sulbactam | 4 | 128 |
|  | + tazobactam | 4 | 128 |
|  | + avibactam | 8 | 128 |
|  | + nacubactam | 8 | 128 |
| tebipenem | alone | 4 | 16 |
|  | + clavulanic acid | 4 | 16 |
|  | + sulbactam | 4 | 16 |
|  | + tazobactam | 4 | 16 |
|  | + avibactam | 8 | 16 |
|  | + nacubactam | 8 | 16 |
| β-lactamase inhibitors |  |  |  |
| clavulanic acid | alone | 512 | > 512 |
| sulbactam | alone | 64 | > 512 |
| tazobactam | alone | 64 | > 512 |
| avibactam | alone | > 512 | > 512 |
| nacubactam | alone | > 512 | > 512 |

MIC, minimal inhibitory concentration

β-lactamase inhibitors were combined to β-lactams with β-lactams at 8 μg/mL.

Table S2 MICs of oral β-lactams alone against *M. avium* ATCC700898 and *M. intracellulare* ATCC13950

|  | MIC (μg/mL) of oral β-lactam against | |
| --- | --- | --- |
| β-lactam | *M. avium* ATCC70089 | *M. intracellulare* ATCC13950 |
| amoxicillin | 4 | 32 |
| cephalexin | 8 | 16 |
| cefaclor | 128 | 128 |
| cefuroxime | 8 | 32 |
| tebipenem | 8 | 16 |
| faropenem | 16 | 32 |

MIC, minimal inhibitory concentration

Table S3 MICs of the combination of two intravenous β-lactams that showed synergy against *M. avium* ATCC700898

| β-lactam A | MIC of β-lactam A (μg/mL) | | β-lactam B | MIC of β-lactam B (μg/mL) | | FIC index |
| --- | --- | --- | --- | --- | --- | --- |
|  | alone | with β-lactam B |  | alone | with β-lactam A |  |
| penicillin G | 4 | 1 | piperacillin | 64 | 16 | 0.5 |
| penicillin G | 4 | 1 | oxacillin | 128 | 32 | 0.5 |
| penicillin G | 4 | 1 | cloxacillin | 128 | 32 | 0.5 |
| penicillin G | 4 | 1 | cefotiam | 32 | 8 | 0.5 |
| penicillin G | 4 | 1 | cefotaxime | 8 | 2 | 0.5 |
| penicillin G | 4 | 1 | ceftazidime | 64 | 16 | 0.5 |
| penicillin G | 4 | 1 | aztreonam | >256 | 64 | 0.38 |
| ampicillin | 4 | 1 | cloxacillin | 128 | 32 | 0.5 |
| ampicillin | 4 | 1 | cefuroxime | 8 | 2 | 0.5 |
| ampicillin | 4 | 1 | cefmetazole | 64 | 16 | 0.5 |
| ampicillin | 4 | 1 | ceftriaxone | 8 | 2 | 0.5 |
| ampicillin | 4 | 1 | ceftazidime | 64 | 16 | 0.5 |
| ampicillin | 4 | 1 | meropenem | 16 | 4 | 0.5 |
| ampicillin | 4 | 1 | imipenem | 32 | 8 | 0.5 |
| cefazolin | 4 | 1 | oxacillin | 128 | 32 | 0.5 |
| cefazolin | 4 | 1 | cloxacillin | 128 | 32 | 0.5 |
| cefazolin | 4 | 1 | nafcillin | 64 | 16 | 0.5 |
| cefazolin | 4 | 1 | cefmetazole | 64 | 16 | 0.5 |
| cefazolin | 4 | 1 | cefoxitin | 32 | 8 | 0.5 |
| cefazolin | 4 | 1 | flomoxef | 16 | 4 | 0.5 |
| cefazolin | 4 | 1 | meropenem | 16 | 4 | 0.5 |
| cefazolin | 4 | 1 | doripenem | 8 | 2 | 0.5 |
| cefazolin | 4 | 1 | imipenem | 32 | 8 | 0.5 |
| ceftriaxone | 8 | 2 | piperacillin | 64 | 16 | 0.5 |
| ceftriaxone | 8 | 2 | oxacillin | 128 | 32 | 0.5 |
| ceftriaxone | 8 | 2 | nafcillin | 64 | 8 | 0.38 |
| ceftriaxone | 8 | 2 | cloxacillin | 128 | 32 | 0.5 |
| ceftriaxone | 8 | 2 | ceftazidime | 64 | 16 | 0.5 |
| ceftriaxone | 8 | 1 | doripenem | 8 | 2 | 0.38 |
| (Table S3 continued) | | | | | | |
| ceftriaxone | 8 | 2 | meropenem | 16 | 4 | 0.5 |
| ceftriaxone | 8 | 2 | imipenem | 32 | 8 | 0.5 |
| cefuroxime | 8 | 2 | cefmetazole | 64 | 16 | 0.5 |
| cefuroxime | 8 | 2 | oxacillin | 128 | 32 | 0.5 |
| cefuroxime | 8 | 2 | cloxacillin | 128 | 16 | 0.38 |
| cefuroxime | 8 | 2 | cefotiam | 32 | 8 | 0.5 |
| cefuroxime | 8 | 2 | meropenem | 16 | 4 | 0.5 |
| cefuroxime | 8 | 2 | imipenem | 32 | 8 | 0.5 |
| cefotaxime | 8 | 2 | oxacillin | 128 | 32 | 0.5 |
| cefotaxime | 8 | 2 | nafcillin | 64 | 16 | 0.5 |
| cefotaxime | 8 | 2 | cloxacillin | 128 | 32 | 0.5 |
| cefotaxime | 8 | 2 | cefmetazole | 64 | 16 | 0.5 |
| doripenem | 8 | 0.25 | oxacillin | 128 | 32 | 0.28 |
| doripenem | 8 | 2 | cefotiam | 32 | 4 | 0.38 |
| doripenem | 8 | 2 | cefoperazone | 16 | 2 | 0.38 |
| doripenem | 8 | 2 | cefmetazole | 64 | 16 | 0.5 |
| doripenem | 8 | 1 | aztreonam | >256 | 32 | 0.19 |
| meropenem | 16 | 0.5 | oxacillin | 128 | 32 | 0.28 |
| meropenem | 16 | 4 | nafcillin | 64 | 8 | 0.38 |
| meropenem | 16 | 4 | cloxacillin | 128 | 32 | 0.5 |
| meropenem | 16 | 4 | cefmetazole | 64 | 16 | 0.5 |
| meropenem | 16 | 2 | cefotiam | 32 | 8 | 0.38 |
| meropenem | 16 | 2 | aztreonam | >256 | 32 | 0.19 |
| flomoxef | 16 | 4 | oxacillin | 128 | 32 | 0.5 |
| flomoxef | 16 | 4 | nafcillin | 64 | 16 | 0.5 |
| flomoxef | 16 | 4 | cefotiam | 32 | 8 | 0.5 |
| flomoxef | 16 | 4 | cloxacillin | 128 | 16 | 0.38 |
| flomoxef | 16 | 2 | aztreonam | >256 | 128 | 0.38 |
| cefepime | 16 | 4 | imipenem | 32 | 8 | 0.5 |
| imipenem | 32 | 8 | cefotiam | 32 | 8 | 0.5 |
| imipenem | 32 | 2 | oxacillin | 128 | 32 | 0.31 |
| imipenem | 32 | 4 | nafcillin | 64 | 16 | 0.38 |
| imipenem | 32 | 8 | ceftazidime | 64 | 16 | 0.5 |
| imipenem | 32 | 4 | aztreonam | >256 | 64 | 0.25 |
| cefotiam | 32 | 4 | piperacillin | 64 | 16 | 0.38 |
| cefotiam | 32 | 8 | oxacillin | 128 | 32 | 0.5 |
| cefotiam | 32 | 4 | nafcillin | 64 | 16 | 0.38 |
| (Table S3 continued) | | | | | | |
| cefotiam | 32 | 8 | cloxacillin | 128 | 32 | 0.5 |
| cefotiam | 32 | 8 | cefoxitin | 32 | 8 | 0.5 |
| cefotiam | 32 | 8 | cefmetazole | 64 | 16 | 0.5 |
| cefotiam | 32 | 8 | ceftazidime | 64 | 16 | 0.5 |
| cefoxitin | 32 | 8 | ceftazidime | 64 | 16 | 0.5 |
| cefoxitin | 32 | 8 | aztreonam | >256 | 32 | 0.31 |
| cefmetazole | 64 | 8 | piperacillin | 64 | 16 | 0.38 |
| cefmetazole | 64 | 8 | oxacillin | 128 | 32 | 0.38 |
| cefmetazole | 64 | 16 | nafcillin | 64 | 16 | 0.5 |
| cefmetazole | 64 | 16 | aztreonam | >256 | 128 | 0.5 |
| piperacillin | 64 | 16 | cloxacillin | 128 | 32 | 0.5 |
| piperacillin | 64 | 16 | ceftazidime | 64 | 16 | 0.5 |

MIC, minimal inhibitory concentration; FIC, fractional inhibitory concentration

Yellow and blue rows indicate the combinations showing the lowest MICs and the lowest FIC indexes, respectively.

Table S4 MICs of the combination of two intravenous β-lactams, not including carbapenems, that showed synergy against *M. intracellulare* ATCC13590

| β-lactam A | MIC of β-lactam A (μg/mL) | | β-lactam B | MIC of β-lactam B (μg/mL) | | FIC index |
| --- | --- | --- | --- | --- | --- | --- |
|  | alone | with β-lactam B |  | alone | with β-lactam A |  |
| cefozopran | 32 | 8 | ampicillin | 64 | 16 | 0.5 |
| cefozopran | 32 | 8 | cefmetazole | 128 | 32 | 0.5 |
| cefozopran | 32 | 8 | ceftazidime | 128 | 32 | 0.5 |
| cefazolin | 64 | 16 | penicillin G | 128 | 32 | 0.5 |
| cefazolin | 64 | 8 | ampicillin | 64 | 16 | 0.38 |
| cefazolin | 64 | 16 | cefmetazole | 128 | 32 | 0.5 |
| cefuroxime | 32 | 8 | penicillin G | 128 | 32 | 0.5 |
| cefuroxime | 32 | 8 | cefmetazole | 128 | 32 | 0.5 |
| cefuroxime | 32 | 8 | cefoxitin | 128 | 32 | 0.5 |
| cefuroxime | 32 | 8 | flomoxef | 64 | 16 | 0.5 |
| ceftriaxone | 32 | 8 | penicillin G | 128 | 16 | 0.38 |
| ceftriaxone | 32 | 8 | cefmetazole | 128 | 32 | 0.5 |
| cefepime | 64 | 16 | cefmetazole | 128 | 32 | 0.5 |
| cefepime | 64 | 16 | flomoxef | 64 | 16 | 0.5 |
| ampicillin | 64 | 16 | cefmetazole | 128 | 16 | 0.38 |
| ampicillin | 64 | 16 | cefotiam | 64 | 16 | 0.5 |
| flomoxef | 64 | 16 | penicillin G | 128 | 32 | 0.5 |
| flomoxef | 64 | 16 | piperacillin | 256 | 64 | 0.5 |
| flomoxef | 64 | 16 | cefmetazole | 64 | 8 | 0.38 |
| flomoxef | 64 | 16 | nafcillin | 64 | 16 | 0.5 |
| flomoxef | 64 | 8 | ceftazidime | 128 | 32 | 0.38 |
| flomoxef | 64 | 16 | cefotiam | 64 | 8 | 0.38 |
| penicillin G | 128 | 32 | piperacillin | 256 | 64 | 0.5 |
| penicillin G | 128 | 16 | nafcillin | 64 | 16 | 0.38 |
| penicillin G | 128 | 32 | cefmetazole | 128 | 32 | 0.5 |
| penicillin G | 128 | 32 | ceftazidime | 128 | 32 | 0.5 |
| ceftazidime | 128 | 32 | piperacillin | 256 | 64 | 0.5 |

MIC, minimal inhibitory concentration; FIC, fractional inhibitory concentration;

Table S5 number of isolates at MIC of oral β-lactam alone against *M. avium* clinical strains

|  |  | number of isolates at MIC (µg/mL) | | | | | | | | | | |
| --- | --- | --- | --- | --- | --- | --- | --- | --- | --- | --- | --- | --- |
| β-lactam | CLR | 0.25 | 0.5 | 1 | 2 | 4 | 8 | 16 | 32 | 64 | 128 | 256 |
| amoxicillin | S |  |  |  | 4 | 9 | 13 | 1 | 2 |  |  |  |
|  | R |  | 1 | 1 | 2 | 6 | 4 | 7 | 5 | 0 | 1 |  |
| cephalexin | S |  |  |  | 6 | 8 | 6 | 7 | 1 | 1 |  |  |
|  | R |  |  | 1 | 3 | 4 | 2 | 8 | 5 | 2 | 1 | 1 |
| faropenem | S |  |  | 1 | 0 | 1 | 5 | 10 | 7 | 3 | 2 |  |
|  | R |  | 3 | 1 | 0 | 2 | 1 | 6 | 5 | 8 | 1 |  |
| cefuroxime | S |  |  |  | 1 | 5 | 11 | 9 | 1 | 2 |  |  |
|  | R |  |  | 1 | 2 | 1 | 5 | 5 | 8 | 4 | 0 | 1 |
| tebipenem | S |  | 1 | 1 | 6 | 14 | 5 | 2 |  |  |  |  |
|  | R | 2 | 2 | 1 | 2 | 6 | 4 | 9 | 1 |  |  |  |

CLR-susceptible: n =29, CLR-resistant: n =27. MIC, minimal inhibitory concentration; CLR, clarithromycin; S, susceptible; R, resistant

Table S6 number of isolates at MIC of oral β-lactam combinations against *M. avium* clinical strains

|  |  |  | number of isolates at MIC (μg/mL) | | | | | | | | | | | | | |
| --- | --- | --- | --- | --- | --- | --- | --- | --- | --- | --- | --- | --- | --- | --- | --- | --- |
| combination | β-lactam | CLR | 0.016 | 0.031 | 0.063 | 0.125 | 0.25 | 0.5 | 1 | 2 | 4 | 8 | 16 | 32 | 64 | 128 |
| cefuroxime amoxicillin | cefuroxime | S |  |  |  |  |  | 4 | 6 | 9 | 5 | 3 | 2 |  |  |  |
|  | cefuroxime | R |  |  |  |  | 1 | 3 | 3 | 2 | 6 | 8 | 3 | 1 |  |  |
|  | amoxicillin | S |  |  |  |  | 1 | 1 | 5 | 12 | 7 | 3 |  |  |  |  |
|  | amoxicillin | R |  |  |  |  | 1 | 1 | 2 | 7 | 6 | 9 | 1 |  |  |  |
| cefuroxime cephalexin | cefuroxime | S |  |  |  |  | 1 | 0 | 7 | 8 | 12 | 1 |  |  |  |  |
|  | cefuroxime | R |  |  |  |  |  | 2 | 5 | 4 | 7 | 4 | 3 | 2 |  |  |
|  | cephalexin | S |  |  |  |  |  | 1 | 6 | 10 | 9 | 2 | 1 |  |  |  |
|  | cephalexin | R |  |  |  |  | 1 | 1 | 3 | 4 | 2 | 8 | 4 | 3 | 0 | 1 |
| cefuroxime faropenem | cefuroxime | S |  |  |  |  |  | 2 | 3 | 7 | 11 | 4 | 2 |  |  |  |
|  | cefuroxime | R |  |  |  |  | 1 | 1 | 1 | 2 | 12 | 4 | 5 | 1 |  |  |
|  | faropenem | S |  |  |  |  |  | 1 | 2 | 7 | 10 | 7 | 1 | 1 |  |  |
|  | faropenem | R |  |  | 1 | 0 | 2 | 2 | 2 | 2 | 9 | 6 | 2 | 1 |  |  |
| tebipenem amoxicillin | tebipenem | S |  |  |  | 1 | 7 | 6 | 7 | 3 | 4 | 1 |  |  |  |  |
|  | tebipenem | R | 2 | 0 | 2 | 0 | 0 | 4 | 4 | 6 | 3 | 6 |  |  |  |  |
|  | amoxicillin | S |  |  |  |  |  | 3 | 3 | 10 | 10 | 1 | 2 |  |  |  |
|  | amoxicillin | R |  |  |  |  | 1 | 3 | 2 | 5 | 6 | 8 | 1 | 1 |  |  |
| tebipenem cephalexin | tebipenem | S |  |  |  |  | 5 | 9 | 7 | 5 | 2 | 1 |  |  |  |  |
|  | tebipenem | R |  |  | 4 | 0 | 4 | 1 | 6 | 3 | 4 | 4 | 1 |  |  |  |
|  | cephalexin | S |  |  |  |  | 1 | 0 | 11 | 5 | 10 | 1 | 1 |  |  |  |
|  | cephalexin | R |  |  |  | 1 | 0 | 0 | 5 | 5 | 1 | 9 | 5 | 1 |  |  |

CLR-susceptible: n =29, CLR-resistant: n =27. MIC, minimal inhibitory concentration; CLR, clarithromycin; S, susceptible; R, resistant

Table S7 MICs used to calculate FIC indexes of oral β-lactam combinations against *M. avium* clinical strain

|  | MIC (μg/mL) | | | | | | | | | | | | | | | | | | | |
| --- | --- | --- | --- | --- | --- | --- | --- | --- | --- | --- | --- | --- | --- | --- | --- | --- | --- | --- | --- | --- |
|  | cefuroxime × amoxicillin | | | | cefuroxime × cephalexin | | | | cefuroxime × faropenem | | | | tebipenem × amoxicillin | | | | tebipenem × cephalexin | | | |
|  | cefuroxime | | amoxicillin | | cefuroxime | | cephalexin | | cefuroxime | | faropenem | | tebipenem | | amoxicillin | | tebipenem | | cephalexin | |
| strain | combo | alone | combo | alone | combo | alone | combo | alone | combo | alone | combo | alone | combo | alone | combo | alone | combo | alone | combo | alone |
| S-Mav01 | 2 | 4 | 2 | 8 | 2 | 4 | 4 | 8 | 2 | 4 | 2 | 8 | 1 | 4 | 2 | 8 | 2 | 4 | 2 | 8 |
| S-Mav02 | 4 | 32 | 8 | 16 | 8 | 32 | 4 | 16 | 8 | 32 | 32 | 128 | 2 | 8 | 8 | 16 | 1 | 8 | 4 | 16 |
| S-Mav03 | 1 | 8 | 2 | 4 | 1 | 8 | 4 | 8 | 2 | 8 | 4 | 16 | 0.5 | 4 | 2 | 4 | 0.5 | 4 | 4 | 8 |
| S-Mav04 | 1 | 8 | 2 | 4 | 4 | 8 | 0.5 | 2 | 2 | 8 | 4 | 16 | 0.25 | 2 | 2 | 4 | 0.25 | 2 | 1 | 2 |
| S-Mav05 | 0.5 | 8 | 2 | 4 | 2 | 8 | 1 | 4 | 2 | 8 | 4 | 16 | 0.25 | 2 | 2 | 4 | 0.5 | 2 | 1 | 4 |
| S-Mav06 | 4 | 16 | 2 | 8 | 2 | 16 | 4 | 32 | 4 | 16 | 8 | 32 | 0.25 | 4 | 4 | 8 | 1 | 4 | 4 | 32 |
| S-Mav07 | 1 | 8 | 2 | 4 | 2 | 8 | 2 | 4 | 2 | 8 | 8 | 16 | 0.25 | 4 | 2 | 4 | 0.5 | 4 | 1 | 4 |
| S-Mav08 | 0.5 | 4 | 1 | 2 | 1 | 4 | 2 | 4 | 1 | 4 | 0.5 | 1 | 0.25 | 0.5 | 0.5 | 2 | 0.25 | 0.5 | 1 | 4 |
| S-Mav09 | 0.5 | 8 | 4 | 8 | 1 | 8 | 8 | 16 | 2 | 8 | 2 | 16 | 1 | 4 | 2 | 8 | 1 | 4 | 2 | 16 |
| S-Mav10 | 4 | 16 | 4 | 8 | 4 | 16 | 2 | 8 | 8 | 16 | 2 | 32 | 2 | 4 | 4 | 8 | 0.5 | 4 | 4 | 8 |
| S-Mav11 | 8 | 16 | 0.25 | 4 | 4 | 16 | 2 | 8 | 4 | 16 | 8 | 32 | 4 | 8 | 1 | 4 | 2 | 8 | 2 | 8 |
| S-Mav12 | 2 | 8 | 2 | 4 | 4 | 8 | 1 | 2 | 4 | 8 | 4 | 16 | 0.5 | 4 | 2 | 4 | 2 | 4 | 1 | 2 |
| S-Mav13 | 8 | 16 | 1 | 8 | 4 | 16 | 4 | 16 | 4 | 16 | 4 | 32 | 4 | 8 | 4 | 8 | 1 | 8 | 4 | 16 |
| S-Mav14 | 4 | 8 | 1 | 8 | 2 | 8 | 2 | 8 | 4 | 8 | 2 | 16 | 1 | 4 | 4 | 8 | 0.5 | 4 | 4 | 8 |
| S-Mav15 | 16 | 64 | 8 | 32 | 4 | 64 | 8 | 16 | 16 | 64 | 16 | 128 | 8 | 16 | 16 | 32 | 8 | 16 | 8 | 16 |
| S-Mav16 | 16 | 64 | 8 | 32 | 4 | 64 | 16 | 64 | 16 | 64 | 4 | 64 | 4 | 16 | 16 | 32 | 4 | 16 | 16 | 64 |
| S-Mav17 | 2 | 8 | 1 | 2 | 1 | 8 | 1 | 2 | 4 | 8 | 1 | 8 | 1 | 2 | 1 | 2 | 0.25 | 2 | 1 | 2 |
| S-Mav18 | 1 | 2 | 0.5 | 2 | 0.25 | 2 | 1 | 2 | 0.5 | 2 | 4 | 8 | 0.5 | 1 | 0.5 | 2 | 0.25 | 1 | 1 | 2 |
| S-Mav19 | 2 | 8 | 2 | 4 | 4 | 8 | 1 | 2 | 4 | 8 | 1 | 8 | 2 | 4 | 2 | 4 | 2 | 4 | 1 | 2 |
| S-Mav20 | 1 | 4 | 2 | 4 | 2 | 4 | 1 | 2 | 2 | 4 | 2 | 8 | 0.25 | 2 | 2 | 4 | 0.25 | 2 | 1 | 2 |
| S-Mav21 | 8 | 16 | 2 | 8 | 4 | 16 | 4 | 8 | 4 | 16 | 8 | 32 | 0.5 | 4 | 4 | 8 | 0.5 | 4 | 4 | 8 |
| S-Mav22 | 2 | 16 | 4 | 8 | 2 | 16 | 2 | 4 | 8 | 16 | 8 | 64 | 1 | 8 | 4 | 8 | 2 | 8 | 2 | 4 |
| S-Mav23 | 2 | 4 | 2 | 8 | 1 | 4 | 2 | 4 | 1 | 4 | 4 | 16 | 0.25 | 2 | 4 | 8 | 1 | 2 | 1 | 4 |
| (Table S7 continued) | | | | | | | | | | | | | | | | | | | | |
| S-Mav24 | 0.5 | 16 | 4 | 8 | 4 | 16 | 4 | 16 | 4 | 16 | 8 | 64 | 0.125 | 4 | 4 | 8 | 1 | 4 | 4 | 16 |
| S-Mav25 | 4 | 16 | 4 | 8 | 4 | 16 | 4 | 16 | 8 | 16 | 4 | 32 | 1 | 4 | 4 | 8 | 0.5 | 4 | 4 | 16 |
| S-Mav26 | 2 | 16 | 2 | 4 | 2 | 16 | 2 | 4 | 4 | 16 | 2 | 16 | 1 | 4 | 2 | 4 | 0.5 | 4 | 2 | 4 |
| S-Mav27 | 2 | 8 | 4 | 8 | 1 | 8 | 2 | 4 | 4 | 8 | 8 | 32 | 4 | 8 | 0.5 | 8 | 4 | 8 | 0.25 | 4 |
| S-Mav28 | 1 | 4 | 1 | 2 | 1 | 4 | 2 | 4 | 0.5 | 4 | 2 | 4 | 0.5 | 2 | 1 | 2 | 0.5 | 2 | 1 | 4 |
| S-Mav29 | 2 | 8 | 4 | 8 | 4 | 8 | 4 | 16 | 1 | 8 | 4 | 16 | 0.5 | 4 | 4 | 8 | 1 | 4 | 4 | 16 |
| R-Mav01 | 8 | 64 | 8 | 32 | 8 | 64 | 32 | 128 | 16 | 64 | 4 | 64 | 8 | 16 | 4 | 32 | 4 | 16 | 32 | 128 |
| R-Mav02 | 2 | 16 | 2 | 4 | 4 | 16 | 2 | 8 | 4 | 16 | 4 | 32 | 2 | 4 | 0.5 | 4 | 0.25 | 4 | 2 | 8 |
| R-Mav03 | 8 | 32 | 8 | 16 | 8 | 32 | 16 | 32 | 8 | 32 | 8 | 64 | 8 | 16 | 8 | 16 | 8 | 16 | 16 | 32 |
| R-Mav04 | 8 | 32 | 4 | 8 | 1 | 32 | 8 | 16 | 8 | 32 | 8 | 64 | 2 | 4 | 4 | 8 | 0.25 | 4 | 8 | 16 |
| R-Mav05 | 0.25 | 2 | 0.25 | 0.5 | 0.5 | 2 | 0.25 | 1 | 1 | 2 | 0.063 | 0.5 | 0.016 | 0.5 | 0.25 | 0.5 | 0.063 | 0.5 | 0.125 | 1 |
| R-Mav06 | 4 | 16 | 4 | 8 | 2 | 16 | 32 | 64 | 4 | 16 | 4 | 16 | 2 | 4 | 2 | 8 | 1 | 4 | 4 | 64 |
| R-Mav07 | 4 | 16 | 4 | 8 | 2 | 16 | 4 | 8 | 4 | 16 | 4 | 16 | 0.5 | 4 | 4 | 8 | 1 | 4 | 2 | 8 |
| R-Mav08 | 1 | 16 | 8 | 16 | 1 | 16 | 8 | 16 | 4 | 16 | 8 | 32 | 4 | 16 | 8 | 16 | 1 | 16 | 8 | 16 |
| R-Mav09 | 4 | 8 | 2 | 4 | 4 | 8 | 1 | 2 | 4 | 8 | 8 | 32 | 2 | 4 | 2 | 4 | 2 | 4 | 1 | 2 |
| R-Mav10 | 0.5 | 1 | 1 | 2 | 0.5 | 1 | 2 | 4 | 0.5 | 1 | 0.25 | 0.5 | 0.063 | 0.25 | 1 | 2 | 0.063 | 0.25 | 2 | 4 |
| R-Mav11 | 4 | 8 | 2 | 4 | 4 | 8 | 1 | 2 | 4 | 8 | 4 | 16 | 1 | 4 | 2 | 4 | 2 | 4 | 1 | 2 |
| R-Mav12 | 8 | 16 | 4 | 16 | 2 | 16 | 8 | 16 | 4 | 16 | 4 | 32 | 4 | 8 | 4 | 16 | 0.25 | 8 | 8 | 16 |
| R-Mav13 | 1 | 4 | 1 | 2 | 2 | 4 | 8 | 32 | 2 | 4 | 0.25 | 0.5 | 0.063 | 0.25 | 0.5 | 2 | 0.063 | 0.25 | 8 | 32 |
| R-Mav14 | 4 | 32 | 8 | 16 | 8 | 32 | 4 | 16 | 16 | 32 | 1 | 16 | 2 | 16 | 8 | 16 | 2 | 16 | 8 | 16 |
| R-Mav15 | 0.5 | 8 | 2 | 4 | 1 | 8 | 2 | 4 | 4 | 8 | 1 | 8 | 0.5 | 2 | 1 | 4 | 0.5 | 2 | 1 | 4 |
| R-Mav16 | 8 | 32 | 8 | 16 | 4 | 32 | 16 | 32 | 4 | 32 | 8 | 32 | 1 | 8 | 8 | 16 | 1 | 8 | 16 | 32 |
| R-Mav17 | 0.5 | 2 | 0.5 | 1 | 1 | 2 | 0.5 | 4 | 0.25 | 2 | 0.5 | 1 | 0.016 | 0.5 | 0.5 | 1 | 0.063 | 0.5 | 1 | 4 |
| R-Mav18 | 2 | 8 | 2 | 4 | 1 | 8 | 2 | 4 | 2 | 8 | 2 | 4 | 0.5 | 2 | 2 | 4 | 1 | 2 | 2 | 4 |
| R-Mav19 | 1 | 8 | 2 | 4 | 4 | 8 | 1 | 2 | 4 | 8 | 0.5 | 4 | 0.5 | 1 | 2 | 4 | 0.25 | 1 | 1 | 2 |
| R-Mav20 | 4 | 32 | 4 | 8 | 4 | 32 | 8 | 16 | 4 | 32 | 4 | 16 | 1 | 8 | 4 | 8 | 4 | 8 | 8 | 16 |
| R-Mav21 | 16 | 256 | 8 | 32 | 32 | 256 | 8 | 16 | 32 | 256 | 4 | 64 | 8 | 16 | 8 | 32 | 8 | 16 | 8 | 16 |
| R-Mav22 | 16 | 64 | 8 | 32 | 16 | 64 | 16 | 32 | 16 | 64 | 2 | 64 | 8 | 16 | 4 | 32 | 1 | 16 | 16 | 32 |
| (Table S7 continued) | | | | | | | | | | | | | | | | | | | | |
| R-Mav23 | 8 | 64 | 16 | 32 | 16 | 64 | 32 | 64 | 16 | 64 | 16 | 128 | 8 | 32 | 16 | 32 | 16 | 32 | 16 | 64 |
| R-Mav24 | 32 | 64 | 4 | 128 | 32 | 64 | 16 | 32 | 16 | 64 | 16 | 64 | 1 | 16 | 32 | 128 | 8 | 16 | 8 | 32 |
| R-Mav25 | 8 | 32 | 8 | 32 | 4 | 32 | 8 | 16 | 8 | 32 | 8 | 64 | 8 | 16 | 8 | 32 | 8 | 16 | 2 | 16 |
| R-Mav26 | 16 | 32 | 2 | 16 | 8 | 32 | 8 | 16 | 4 | 32 | 4 | 16 | 2 | 16 | 8 | 16 | 4 | 16 | 8 | 16 |
| R-Mav27 | 8 | 32 | 8 | 16 | 16 | 32 | 128 | 256 | 8 | 32 | 32 | 64 | 4 | 8 | 8 | 16 | 4 | 8 | 16 | 256 |

S-Mav, CAM-susceptible *M. avium* (n =29); R-Mav, CAM-resistant *M. avium* (n =27); combo, in combination with the other β-lactam

Table S8 number of isolates at MIC of intravenous β-lactams alone against *M. intracellulare* clinical strains

|  |  | number of isolates at MIC (µg/mL) | | | | | | | | | | MIC_50_ | MIC_90_ |
| --- | --- | --- | --- | --- | --- | --- | --- | --- | --- | --- | --- | --- | --- |
| β-lactam | CLR | 0.5 | 1 | 2 | 4 | 8 | 16 | 32 | 64 | 128 | 256 |  |  |
| doripenem | S | 1 | 0 | 0 | 1 | 2 | 3 | 4 | 9 | 5 |  | 64 | 128 |
|  | R |  | 1 | 0 | 0 | 0 | 1 | 1 | 0 | 1 |  | - | - |
| meropenem | S | 1 | 0 | 0 | 1 | 2 | 1 | 5 | 9 | 6 |  | 64 | 128 |
|  | R |  | 1 | 0 | 0 | 0 | 1 | 1 | 0 | 1 |  | - | - |
| ceftriaxone | S |  |  |  |  | 2 | 2 | 6 | 5 | 9 | 1 | 64 | 128 |
|  | R |  |  |  | 1 | 1 | 0 | 1 | 0 | 0 | 1 | - | - |

CLR-susceptible: n =25, CLR-resistant: n = 4. MIC, minimal inhibitory concentration; CLR, clarithromycin; S, susceptible; R, resistant

Table S9 number of isolates at MIC of intravenous β-lactam combinations against *M. intracellulare* clinical strains

|  |  |  | number of isolates at MIC (μg/mL) | | | | | | | | | | |
| --- | --- | --- | --- | --- | --- | --- | --- | --- | --- | --- | --- | --- | --- |
| combination | β-lactam | CLR | 0.125 | 0.25 | 0.5 | 1 | 2 | 4 | 8 | 16 | 32 | 64 | 128 |
| ceftriaxone doripenem | ceftriaxone | S |  |  |  |  | 3 | 1 | 4 | 8 | 4 | 4 | 1 |
|  | ceftriaxone | R |  |  |  | 1 | 0 | 1 | 1 | 0 | 1 |  |  |
|  | doripenem | S | 1 | 0 | 1 | 1 | 3 | 3 | 5 | 5 | 5 | 1 |  |
|  | doripenem | R | 1 | 0 | 1 | 0 | 0 | 1 | 0 | 0 | 1 |  |  |
| ceftriaxone meropenem | ceftriaxone | S |  |  |  |  | 2 | 3 | 3 | 6 | 7 | 3 | 1 |
|  | ceftriaxone | R |  |  |  | 1 | 0 | 1 | 1 | 0 | 0 | 1 |  |
|  | meropenem | S | 1 | 0 | 1 | 1 | 3 | 3 | 5 | 5 | 5 | 1 |  |
|  | meropenem | R |  | 1 | 0 | 1 | 0 | 1 | 0 | 0 | 1 |  |  |

CLR-susceptible: n =25, CLR-resistant: n = 4. MIC, minimal inhibitory concentration; CLR, clarithromycin; S, susceptible; R, resistant.

Table S10 MICs used to calculate FIC indexes of intravenous β-lactam combinations against *M. intracellulare* clinical isolates

| strain | MIC (μg/mL) | | | | | | | |
| --- | --- | --- | --- | --- | --- | --- | --- | --- |
|  | ceftriaxone × doripenem | | | | ceftriaxone × meropenem | | | |
|  | ceftriaxone | | doripenem | | ceftriaxone | | meropenem | |
|  | combo | alone | combo | alone | combo | alone | combo | alone |
| S-Min01 | 64 | 128 | 32 | 128 | 64 | 128 | 16 | 128 |
| S-Min02 | 8 | 64 | 8 | 16 | 32 | 64 | 1 | 16 |
| S-Min03 | 32 | 64 | 4 | 32 | 16 | 64 | 8 | 32 |
| S-Min04 | 64 | 128 | 8 | 64 | 32 | 128 | 32 | 128 |
| S-Min05 | 16 | 32 | 16 | 64 | 16 | 32 | 16 | 128 |
| S-Min06 | 2 | 16 | 8 | 16 | 4 | 16 | 1 | 8 |
| S-Min07 | 32 | 128 | 16 | 64 | 64 | 128 | 8 | 64 |
| S-Min08 | 2 | 8 | 1 | 4 | 2 | 8 | 1 | 4 |
| S-Min09 | 16 | 64 | 4 | 64 | 16 | 64 | 4 | 64 |
| S-Min10 | 16 | 128 | 32 | 64 | 32 | 128 | 16 | 64 |
| S-Min11 | 16 | 64 | 16 | 64 | 16 | 64 | 16 | 64 |
| S-Min12 | 64 | 128 | 0.5 | 8 | 32 | 128 | 2 | 8 |
| S-Min13 | 32 | 128 | 64 | 128 | 32 | 128 | 64 | 128 |
| S-Min14 | 128 | 256 | 16 | 128 | 128 | 256 | 16 | 128 |
| S-Min15 | 16 | 32 | 2 | 32 | 8 | 32 | 8 | 32 |
| S-Min16 | 4 | 16 | 2 | 8 | 4 | 16 | 8 | 32 |
| S-Min17 | 16 | 128 | 32 | 64 | 32 | 128 | 32 | 64 |
| S-Min18 | 8 | 32 | 4 | 16 | 4 | 32 | 16 | 32 |
| S-Min19 | 8 | 32 | 16 | 64 | 8 | 32 | 16 | 64 |
| S-Min20 | 32 | 128 | 32 | 128 | 32 | 128 | 16 | 128 |
| S-Min21 | 64 | 128 | 8 | 64 | 64 | 128 | 16 | 64 |
| S-Min22 | 16 | 64 | 32 | 128 | 16 | 64 | 8 | 64 |
| S-Min23 | 16 | 32 | 2 | 32 | 16 | 32 | 2 | 32 |
| S-Min24 | 8 | 32 | 8 | 32 | 8 | 32 | 16 | 64 |
| S-Min25 | 2 | 8 | 0.125 | 0.5 | 2 | 8 | 0.125 | 0.5 |
| R-Min01 | 8 | 32 | 4 | 32 | 8 | 32 | 4 | 32 |
| R-Min02 | 32 | 256 | 32 | 128 | 64 | 256 | 32 | 128 |
| R-Min03 | 1 | 4 | 0.125 | 1 | 1 | 4 | 0.25 | 1 |
| R-Min04 | 4 | 8 | 0.5 | 16 | 4 | 8 | 1 | 16 |

S-Min, CAM-susceptible *M. intracellulare* (n =25); R-Min, CAM-resistant *M. intracellulare* (n =4)

combo, in combination with the other β-lactam

Fig. S1 FIC indexes of intravenous β-lactam combinations against *M. intracellulare* clinical strains

Each point indicates the FIC index on each strain, and each bar shows the median of the FIC indexes. Green points and bars represent CLR-sensitive strains (n =25) and orange ones represent CLR-resistant strains (n =4). FIC index ≤ 0.5 was judged as synergistic and 0.5 < FIC index ≤ 1.0 as additive.

Table S11 List of the antibacterial agents used in this study

| **class** | | **Antibaiotics** | **Source of purchase** |
| --- | --- | --- | --- |
| **oral β-lactams** | penicillins | amoxicillin | Tokyo Chemical Industry (Tokyo, Japan) |
|  | cephalosporins | cephalexin | Tokyo Chemical Industry (Tokyo, Japan) |
|  |  | cefaclor | Tokyo Chemical Industry (Tokyo, Japan) |
|  |  | cefuroxime | Tokyo Chemical Industry (Tokyo, Japan) |
|  | carbapenems | tebipenem | Meiji Seika Pharma Co., Ltd. (Tokyo, Japan) |
|  | penems | faropenem | AK Scientific (Union City, CA, USA) |
| **intravenous β-lactams** | penicillins | penicillin G | Tokyo Chemical Industry (Tokyo, Japan) |
|  |  | ampicillin | Tokyo Chemical Industry (Tokyo, Japan) |
|  |  | piperacillin | Tokyo Chemical Industry (Tokyo, Japan) |
|  |  | oxacillin | Tokyo Chemical Industry (Tokyo, Japan) |
|  |  | nafcillin | Cayman Chemical (Ann Arbor, Michigan, USA) |
|  |  | cloxacillin | Tokyo Chemical Industry (Tokyo, Japan) |
|  | cephalosporins | cefazolin | Tokyo Chemical Industry (Tokyo, Japan) |
|  |  | cefotiam | Sigma-Aldrich (Tokyo, Japan) |
|  |  | cefoxitin | Tokyo Chemical Industry (Tokyo, Japan) |
|  |  | cefuroxime | Tokyo Chemical Industry (Tokyo, Japan) |
|  |  | cefmetazole | Tokyo Chemical Industry (Tokyo, Japan) |
|  |  | ceftriaxone | Tokyo Chemical Industry (Tokyo, Japan) |
|  |  | cefotaxime | Cayman Chemical (Ann Arbor, Michigan, USA) |
|  |  | cefoperazone | Cayman Chemical (Ann Arbor, Michigan, USA) |
|  |  | ceftazidime | Tokyo Chemical Industry (Tokyo, Japan) |
|  |  | cefepime | MedChem Express, (Monmouth Junction, NJ, USA) |
|  |  | cefozopran | AmBeed (Arlington Hts, IL, USA) |
|  | oxacephems | flomoxef | Sigma-Aldrich (Tokyo, Japan) |
|  | monobactams | aztreonam | Tokyo Chemical Industry (Tokyo, Japan) |
|  | carbapenems | imipenem | MedChem Express, (Monmouth Junction, NJ, USA) |
|  |  | meropenem | Tokyo Chemical Industry (Tokyo, Japan) |
|  |  | doripenem | Tokyo Chemical Industry (Tokyo, Japan) |
| **β-lactamase**  **inhibitors** | | clavulanic acid | Cmobi-Blocks (San Diego, CA, USA) |
|  |  | sulbactam | Tokyo Chemical Industry (Tokyo, Japan) |
|  |  | tazobactam | Tokyo Chemical Industry (Tokyo, Japan) |
|  |  | avibactam | AmBeed (Arlington Hts, IL, USA) |
|  |  | nacbactam | Meiji Seika Pharma Co., Ltd. (Tokyo, Japan) |
| **others** | | clarithromycin | Tokyo Chemical Industry (Tokyo, Japan) |
|  |  | moxifloxacin | Tokyo Chemical Industry (Tokyo, Japan) |
|  |  | linezolid | Tokyo Chemical Industry (Tokyo, Japan) |
